# Endocytic pathways mediating oligomeric Aβ42 neurotoxicity

**DOI:** 10.1186/1750-1326-5-19

**Published:** 2010-05-17

**Authors:** Chunjiang Yu, Evelyn Nwabuisi-Heath, Kevin Laxton, Mary Jo LaDu

**Affiliations:** 1 Department of Anatomy and Cell Biology, University of Illinois at Chicago, Chicago, IL 60612 USA

## Abstract

**Background:**

One pathological hallmark of Alzheimer's disease (AD) is amyloid plaques, composed primarily of amyloid-β peptide (Aβ). Over-production or diminished clearance of the 42 amino acid form of Aβ (Aβ42) in the brain leads to accumulation of soluble Aβ and plaque formation. Soluble oligomeric Aβ (oAβ) has recently emerged to be as a likely proximal cause of AD.

**Results:**

Here we demonstrate that endocytosis is critical in mediating oAβ42-induced neurotoxicity and intraneuronal accumulation of Aβ. Inhibition of clathrin function either with a pharmacological inhibitor, knock-down of clathrin heavy chain expression, or expression of the dominant-negative mutant of clathrin-assembly protein AP180 did not block oAβ42-induced neurotoxicity or intraneuronal accumulation of Aβ. However, inhibition of dynamin and RhoA by expression of dominant negative mutants reduced neurotoxicity and intraneuronal Aβ accumulation. Pharmacologic inhibition of the dynamin-mediated endocytic pathway by genistein also reduced neurotoxicity.

**Conclusions:**

These data suggest that dynamin-mediated and RhoA-regulated endocytosis are integral steps for oligomeric Aβ42-induced neurotoxicity and intraneuronal Aβ accumulation.

## Background

Amyloid-β peptide (Aβ) is believed to be a causative agent underlying the pathological mechanism for Alzheimer's disease, the major form of dementia in the elderly [1]. The levels of soluble Aβ species appear to correlate with disease progression [2-12]. Evidence points to soluble oligomeric Aβ (oAβ) as the assembly form of the peptide that is likely the proximal cause in AD [13-24], leading to synaptic dysfunction and eventual neuron loss in the vulnerable regions of AD brains (for recent review [25]). Extracellular oAβ has been proposed to bind the cell surface, leading to functional disruption of NMDAR [26,27] and AMPAR [28,29], and activation of caspases [30].

In addition to extracellular Aβ, Aβ accumulates inside neurons. Intraneuronal Aβ accumulation has been identified in Down syndrome and AD patients, amyloid precursor protein (APP) and PS1 Presenilin 1 transgenic mice, and cultured cells [31-48]. In AD patients, intraneuronal Aβ42 accumulation appears in vulnerable brain regions prior to extracellular amyloid formation and accumulates with aging [31-37,39,44,45,49-52]. In addition, synaptic dysfunction occurs prior to, or in the absence of, amyloid plaques in both AD and APP transgenic mouse brains [9,53-56]. Studies using triple transgenic mice demonstrated that intraneuronal Aβ causes the onset of early AD-related cognitive deficits [43,57,58]. Intriguingly, clearance of intraneuronal Aβ by immunotherapy rescued early cognitive deficits, prior to changes in plaque density. Intraneuronal Aβ and cognitive deficits re-emerged with the subsequent withdrawal of immunotherapy [58,59]. These observations support the hypothesis that intraneuronal Aβ accumulation may be one of the initial steps in a cascade of events leading to AD [60,61]. Neurons internalize and accumulate exogenous Aβ [62-65]. Intraneuronal Aβ could be viewed as compromised clearance of extracellular soluble Aβ by neurons, and excessive accumulation of intraneuronal Aβ could lead to cellular organelle dysfunction and eventual neuron death. For example, intraneuronal Aβ was reported to activate caspase 6 leading to neuronal apoptosis [66]. We recently demonstrated that intracellular oAβ42 can activate casein kinase-2, causing inhibition of fast axonal transport [67].

Neurons, like many other cell types, have several major endocytic pathways, including clathrin-dependent, caveolae-dependent, and clathrin- and caveolar-independent pathways. However, the specific endocytic pathways involved in oAβ-uptake and neurotoxicity remain unclear. Using complementary approaches of pharmacological inhibition, genetic manipulation by over-expressing dominant-negative mutants and gene knock-down, we provide data that show that the endocytosis of oAβ42 is linked to neurotoxicity via a dynamin-dependent and RhoA-mediated endocytic pathway *in vitro*.

We previously established a homogenous preparation of oAβ42 [19] that causes neurotoxicity in co-cultures of primary neurons and glia, as well as Neuro-2A cells (N2A) [18,68,69]. This oAβ42 preparation also inhibits LTP [70], causes cognitive deficits [71], disrupts fast axonal transport [67], and induces neuroinflammation [72]. Here we focus mainly on endocytic pathways in relation to oAβ42 toxicity in N2A cells.

## Results

### Clathrin-dependent endocytic pathway is not involved in oAβ42 mediated toxicity

**Figure 1:**
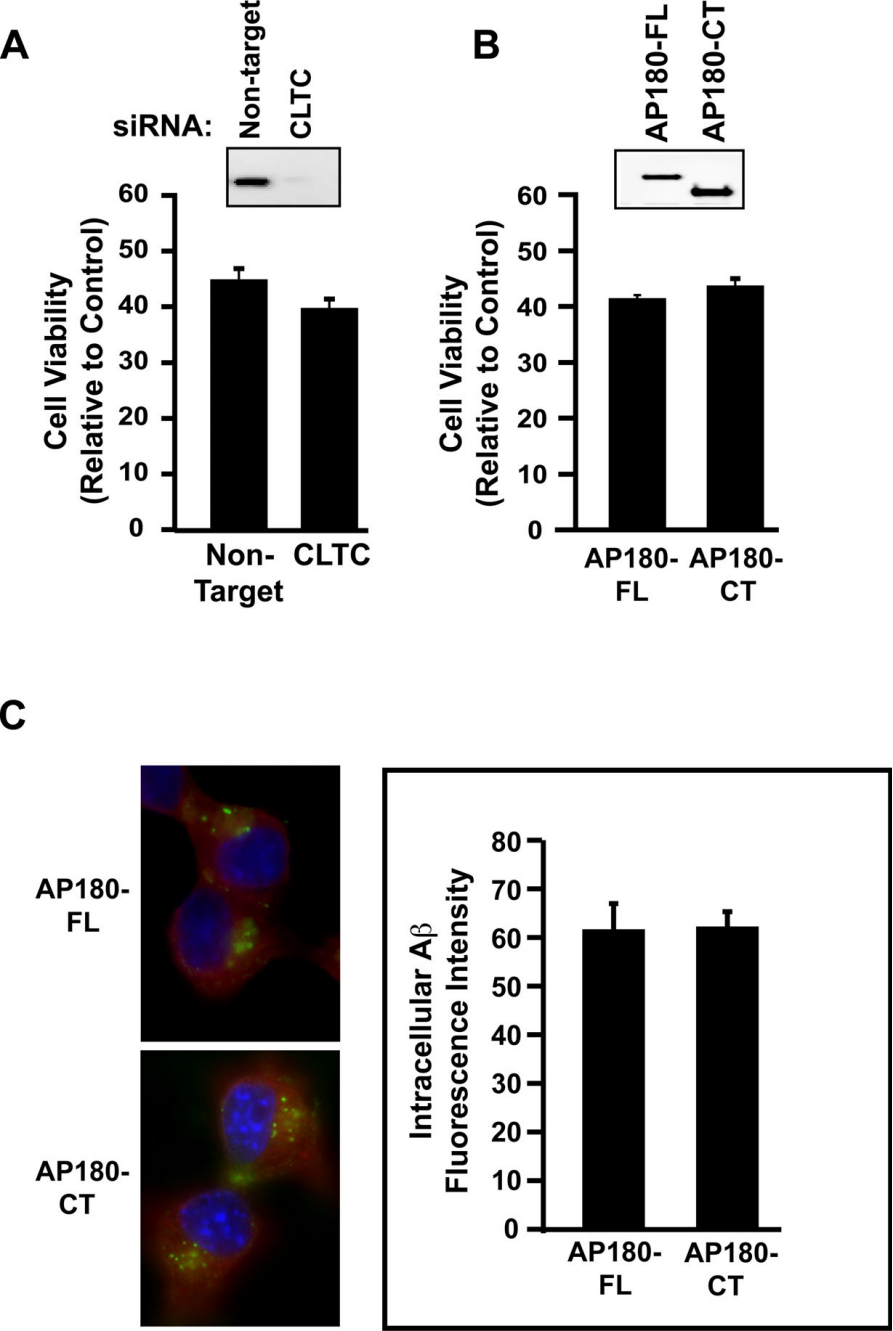
**The clathrin mediated endocytic pathway is not involved in oligomeric Aβ42-induced neurotoxicity**. **A**. N2A cells were transiently transfected with siRNA for clathrin heavy chain, treated with 10 μM oAβ42 for 24 hours and assayed for neurotoxicity as detected with an ATP-based luminescence cell viability assay (CellTiter-Glo, Promega); no difference with treatment. **Inset**, clathrin heavy chain levels were characterized with clathrin heavy chain antibody (Sigma) by Western blot analysis with an equal amount of lysates from cells transfected with non-targeted siRNA and CLTC siRNA. **B**. N2A cells were transiently transfected with wild type AP180-FL or dominant-negative AP180-CT construct, treated with 10 μM oAβ42 for 24 hours, and assayed for neurotoxicity; no difference with treatment. **Inset**, AP180-CT was detected with Flag antibody (Sigma) by Western blot analysis with an equal amount of lysate from cells transfected with AP180-FL and AP180-CT. **C**. N2A cells were transiently transfected with AP180-FL or AP180-CT. 48 hours post-transfection, cells were treated with 10 μM oAβ42 for 30 minutes, fixed and stained for Aβ with Aβ42 specific antibody (Invitrogen, green). AP180-FL and AP180-CT mutant transfected cells were identified by anti-Flag antibody (red). Nuclei appear blue as detected by DAPI staining. Cells were individually outlined and mean fluorescence intensity of Aβ signals was quantified with NIH image software. There were similar levels of Aβ accumulation in these transfected cells.

As accumulation of intracellular Aβ42 accompanies neurotoxicity, we wanted to determine whether blocking specific endocytic pathways would inhibit neurotoxicity. We used several approaches to examine clathrin-mediated endocytosis, a major endocytic pathway. First, to directly target clathrin, we transiently transfected N2A cells with siRNA specifically targeting mouse clathrin heavy chain. Western blot analysis showed that siRNA substantially reduced clathrin protein levels, in comparison to non-target siRNA control (Figure 1A inset). Further, the knock-down of clathrin inhibited transferrin uptake in these transfected cells (data not shown). However, clathrin siRNA failed to block oAβ42 toxicity, similar to the non-targeting siRNA control (Figure 1A). Second, we expressed a dominant-negative mutant of the neuron-specific clathrin-assembly protein AP180, AP180-CT. The construct contains the clathrin-binding domain at C-terminal region of AP180, and its expression is known to inhibit clathrin-mediated endocytosis [73,74]. We transiently transfected N2A cells with AP180 full-length wild type or a dominant-negative mutant AP180-CT. Transiently transfected N2A cells expressed AP180 and AP180-CT as detected by Western blot analysis (Figure 1B inset). However, the AP180-CT mutant did not inhibit cell toxicity induced by oAβ42, similar to the wild type control (Figure 1B). Furthermore, in both AP180 and AP180-CT mutant transfected cells, there were similar levels of intracellular Aβ accumulation as detected by immunofluorescence quantitation (Figure 1C). Third, chlorpromazine, a cationic amphiphilic drug that inhibits the formation of clathrin-coated pits [75], was tested. While this compound is toxic to N2A cells at high concentration, treatment with 2 mM chlorpromazine retained 90% cell viability. Again, this treatment failed to block oAβ42 neurotoxicity (data not shown). These combined results strongly suggest that under our experimental conditions, the clathrin-dependent endocytic pathway does not participate in oAβ42-induced neurotoxicity.

### Dynamin mediates oAβ42 neurotoxicity and intracellular accumulation

**Figure 2:**
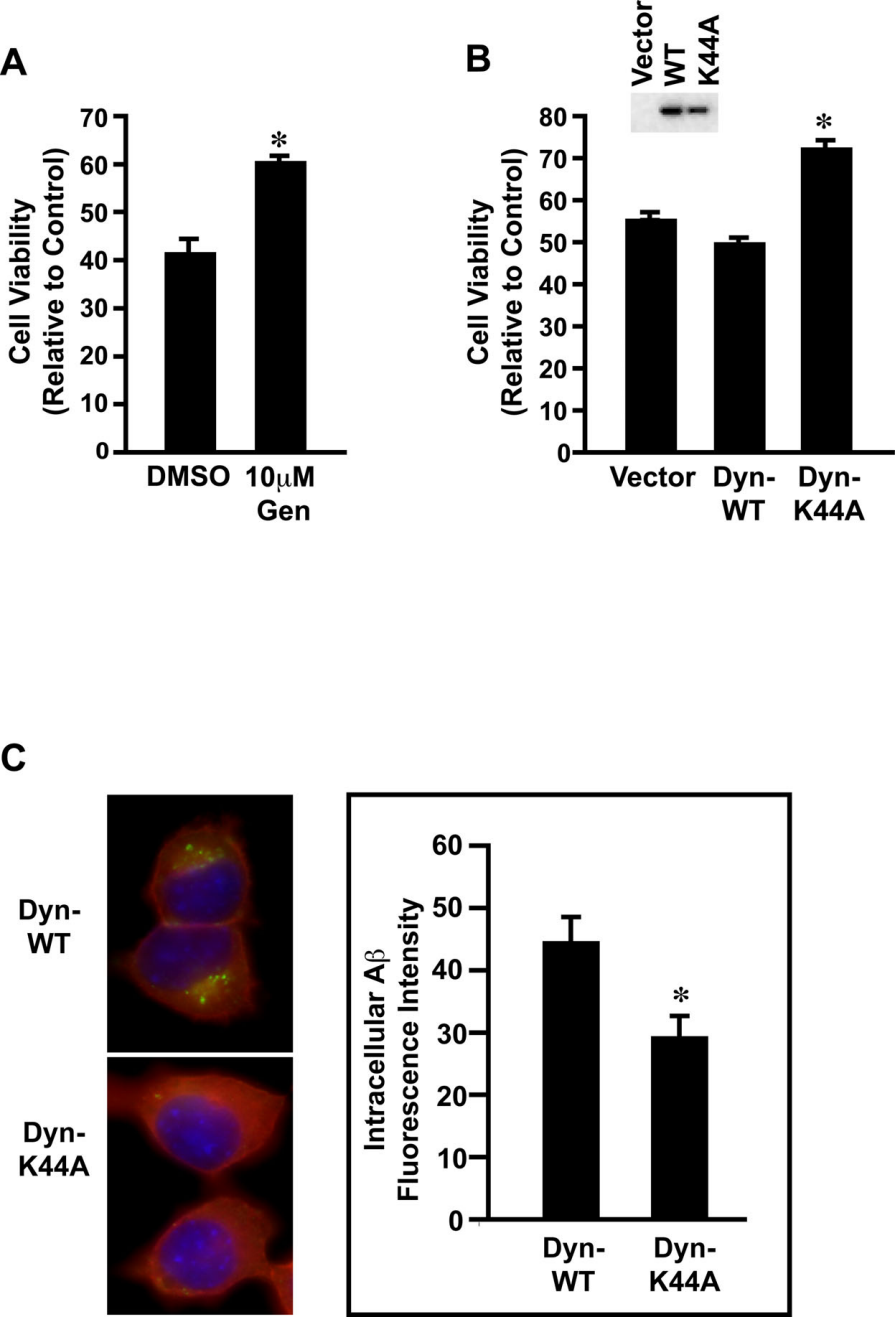
**Dynamin mediates oligomeric Aβ42-induced neurotoxicity**. **A**. N2A cells were pre-treated ± 10 mM genistein for 1 hour, treated with 10 μM oAβ42 for 24 hours ± genistein, and assayed for neurotoxicity as described in Figure Legend 1. Significant difference (*p *< 0.01) between cells ± genistein is indicated by an asterisk (*). **B**. N2A cells were transiently transfected with dynamin wild type, dominant-negative K44A mutant expression plasmids, or vector control; treated with 10 μM oAβ42 for 24 hours and assayed for neurotoxicity. Significant difference (*p *< 0.01) between cells ± dominant-negative K44A mutant are indicated by an asterisk (*). Inset, expression levels of c-myc tagged dynamin were characterized by anti-myc antibody with Western blot analysis with an equal amount of lysate from cells transfected with vector, dynamin wild type, or dynamin dominant-negative K44A mutant. **C**. N2A cells were transiently transfected with dynamin wild type or dominant-negative K44A mutant expression plasmids. 48 hours post-transfection, cells were treated with 10 μM oAβ42 for 30 minutes, and stained for Aβ with Aβ42 specific antibody (Invitrogen, green). Transfected cells were identified by anti-myc antibody (Abcam, red). Nuclei appear blue as detected by DAPI staining. Cells were individually outlined and mean fluorescence intensity of Aβ signals were quantified with NIH image software. Significant difference in Aβ levels (*p *< 0.01) between cells with dynamin wild type and K44A mutant is indicated by an asterisk (*).

Dynamin mediates both clathrin-dependent and -independent endocytosis (for review, [76]). We first used two pharmacological inhibitors to block the dynamin-dependent endocytic pathway, genistein (a general tyrosine kinase inhibitor) and PP2 (a Src family tyrosine kinase inhibitor). As shown in Figure 2A, genistein significantly inhibited oAβ42-induced toxicity. The result was consistent with previous reports that this agent protected from Aβ induced toxicity in cultured hippocampal neurons [77] and SH-SY5Y cells [78]. PP2 pre-treatment also decreased Aβ42 toxicity though to a lesser extent than genistein (data not shown). In addition, we used a dominant-negative dynamin mutant K44A, an established reagent to specifically abolish dynamin function [79]. Both dynamin K44A mutant and wild type proteins were expressed in the transfected cells, as determined by Western blot analysis (Figure 2B inset). With oAβ42 treatment, the K44A mutant inhibited neurotoxicity compared to wild type dynamin (Figure 2B). Thus, the prediction would be that blocking dynamin mediated oAβ42 endocytosis would decrease intracellular Aβ accumulation. N2A cells were transfected with dynamin wild type or mutant K44A. Treatment of these transfected cells with oAβ42 resulted in significantly less intracellular Aβ in dynamin mutant cells compared to dynamin wild type cells (Figure 2C). These data collectively support a role for dynamin in oAβ42 endocytosis and neurotoxicity.

### RhoA regulates oAβ42 neurotoxicity and intracellular accumulation

**Figure 3:**
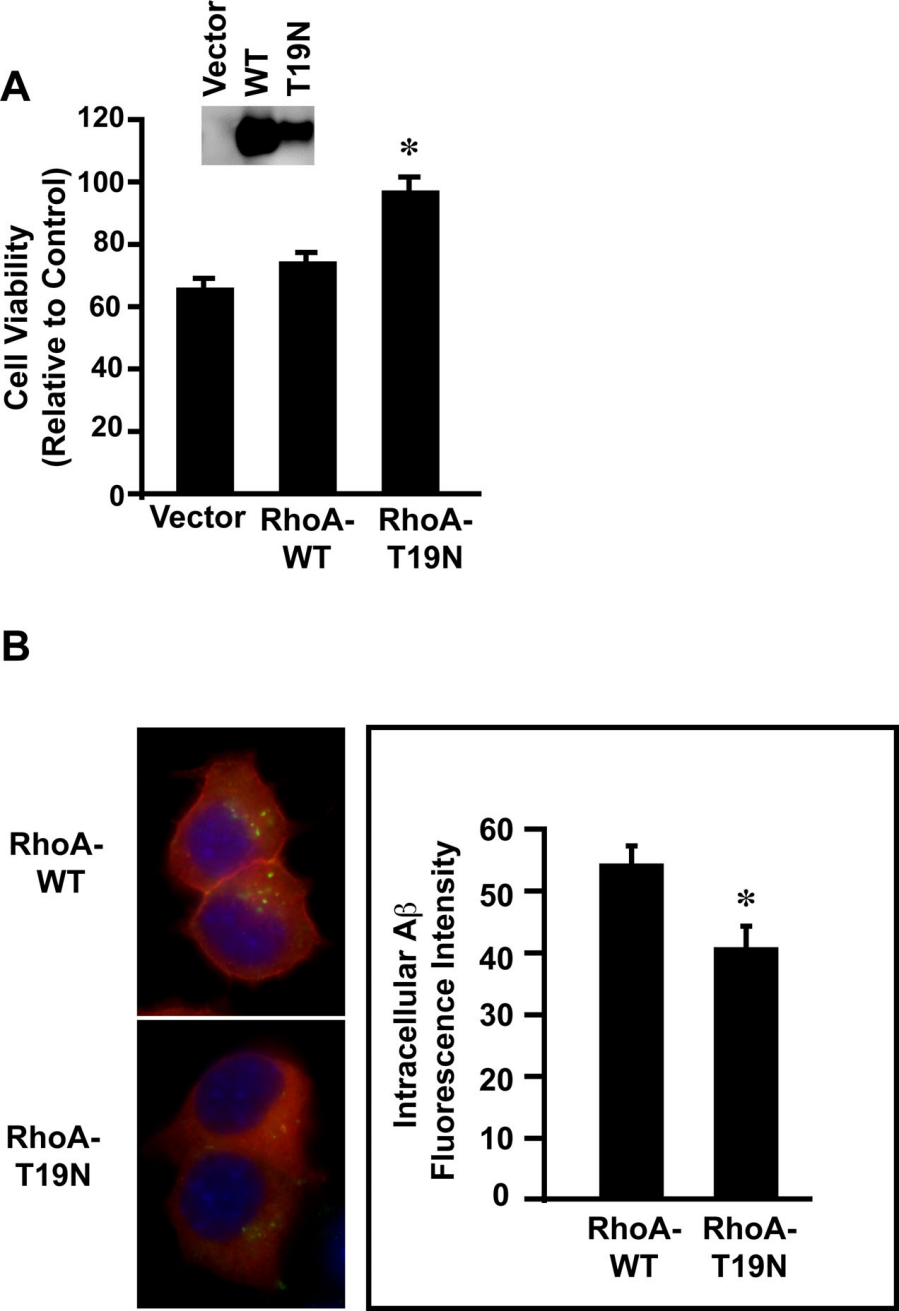
**RhoA mediates oligomeric Aβ42-induced neurotoxicity**. **A**. N2A cells were transiently transfected with RhoA wild type, dominant-negative T19N mutant, or vector control; treated with oAβ42 for 24 hours; and assayed for neurotoxicity as described in Figure Legend 1. Significant difference (*p *< 0.01) between cells ± RhoA dominant-negative T19N mutant are indicated by an asterisk (*). Inset, expression levels of HA-taged RhoA were characterized by anti-HA antibody (Roche) with Western blot analysis with an equal amount of lysate from cells transfected vector, RhoA wild type or RhoA dominant-negative T19N mutant. **B**. N2A cells were transiently trasfected with RhoA wild type or dominant-negative T19N mutant expression plasmids. 48 hours post-transfection, cells were treated with 10 μM oAβ42 for 30 minutes, fixed and stained for Aβ with Aβ42 specific antibody (Invitrogen, green). RhoA wild type or T19N mutant transfected cells were identified by anti-HA antibody (red). Nuclei appear blue as detected by DAPI staining. Cells were individually outlined and mean fluorescence intensity of Aβ signals were quantified with NIH image software. Significant difference (*p *< 0.01) between cells transfected with RhoA wild type and T19N mutant is indicated by an asterisk (*).

We next determined the role of RhoA in oAβ42 neurotoxicity and endocytosis. The small GTPase RhoA regulates the clathrin-independent endocytic pathways [76]. For these experiments, we transiently transfected N2A cells with vector alone, RhoA wild type or a dominant-negative RhoA mutant T19N [80]. Western blot analysis confirmed expression of RhoA and T19N proteins (Figure 3A inset). In cells treated with oAβ42, the T19N RhoA mutant significantly protected cells from oAβ42-induced neurotoxicity compare to RhoA wild type (Figure 3A). To confirm that RhoA is involved in oAβ42 endocytosis, we assessed accumulation of intracellular Aβ by treating these transfected cells oAβ42. Significantly less Aβ was detected in RhoA mutant positive cells compared to RhoA wild type cells (Figure 3B). These results suggest that RhoA is involved in oAβ42 endocytosis and neurotoxicity.

**Figure 4:**
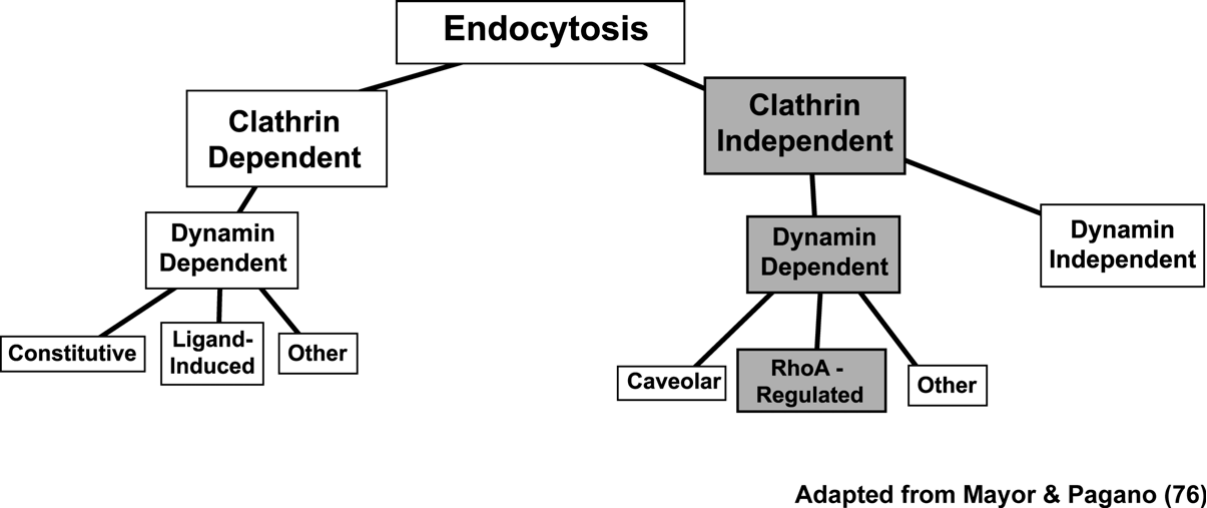
**Model for endocytic pathways mediating oligomeric Aβ42-induced neurotoxicity**. The endocytic pathways for oAβ neurotoxicity and intracellular accumulation is clathrin independent, but dynamin dependent. The pathways are further regulated by small GTPase RhoA. This figure is adapted from Mayor and Pagano [76].

Taken together, these data strongly suggest that endocytosis is critical for oAβ42-induced neurotoxicity. This process is dependent on dynamin, but not clathrin, and further regulated by RhoA (Figure 4).

## Discussion

In recent years, it has become increasingly clear that soluble oAβ plays an essential role in the neuronal loss characteristic of AD pathology. Soluble oAβ could mediate neuronal dysfunction extracellularly by binding to cell surface receptors and disturbing downstream signaling pathways, leading to disruption of LTP and LTD, and eventual neuronal death. Alternatively, soluble oAβ toxicity could arise from intraneuronal accumulation as a result of impaired exocytosis or failed clearance following endocytosis. The importance of endocytosis in AD is underscored by a recent report identifying genetic variances in phosphatidylinositol-binding clathrin assembly protein (PICALM) associated with late onset AD [81]. PICALM facilitates endocytosis in hippocampal neurons and thus could play a role in Aβ clearance in the brain [82]. However, the mechanisms underlying binding and subsequent signalling pathways or endocytosis leading to Aβ intracellular accumulation remain poorly understood.

Although a major endocytic pathway in neurons is clathrin-dependent [83], we show by three complementary approaches that inhibition of this pathway did not inhibit oAβ42 neurotoxicity (Figure 1). A reduced level of AP180 has been reported in AD patient brains [84]. Our data that AP180 did not mediate Aβ toxicity suggests that AP180 could potentially regulate trafficking of proteins/enzymes involved in Aβ production [85].

There is increasing evidence of clathrin- and caveolin-independent pathways mediating ligand-induced endocytosis [76,86]. The large GTPase dynamin is involved in both clathrin-dependent and -independent pathways [76]. Our results suggest an important role for dynamin in oAβ42-induced neurotoxicity and intraneuronal Aβ accumulation. Interestingly, clathrin-independent but dynamin-dependent endocytosis was required for Aβ internalization in sympathetic neurons *in vitro *[87]. The small monomeric GTPase RhoA regulates other clathrin-independent pathways, such as IL2-receptor endocytosis, [88]. Our data show RhoA regulates oAβ42 endocytosis and neurotoxicity. The role of RhoA in oAβ42-induced neurotoxicity is further supported by recent reports of potential roles for this GTPase in AD. For example, RhoA had an altered subcellular localization in both AD and APP transgenic Tg2576 mouse brains [89]. Further, RhoA levels increased specifically around amyloid plaques in these models [90].

As neurons do not express caveolin-1, the principal structural protein in caveolae, and do not have caveolae structure [91], we did not pursue this pathway. Another possible route for Aβ uptake is pinocytosis. Aβ40 directly conjugated with fluorescein was taken up by neurons via diffusion in a non-saturable, energy-independent process [92]. In our experiments, ATP levels were used as a measurement of neurotoxicity, precluding results based on energy independence. More importantly, while we have been able to consistently label oligomers with Alexa-488 after formation and maintain conformational stability, we are unable to prepare consistent oligomeric conformations using pre-labeled fluorescein-Aβ42 [65]. In addition, comparison between Aβ40 and 42 is problematic.

Our data (summarized in the schematic shown in Figure 4) show RhoA and dynamin-dependent steps involved in oAβ42 neurotoxicity and intracellular Aβ accumulation.

## Conclusions

Our experiments identify the initial steps of endocytosis required for oAβ42-induced neurotoxicity and intracellular Aβ accumulation. Specifically, Aβ-induced neurotoxicity is dynamin-dependent and RhoA-regulated, but clathrin-independent. Further studies will be needed to identify potential steps in the endocytic pathways as therapeutic targets in AD.

## Methods

### Materials

Recombinant Aβ42 was purchased from rPeptide (Bogart, GA). Hexafluoroisopropanol (HFIP) and anhydrous dimethyl sulfoxide (DMSO) were purchased from Sigma-Aldrich. Phenol-red free Ham's F12 media was obtained from Promocell (Heidelberg, Germany) and supplemented with L-glutamine (146 mg/L) prior to use. Genistein and chlorpromazine were purchased from Sigma. PP2 was purchased from EMD Biosciences.

### Oligomer formation conditions

Oligomer preparations of Aβ42 were formed according to our previously established protocols [18,19]. Briefly, following evaporation of HFIP in a fume hood overnight, the resulting Aβ42 peptide film was stored desiccated at -20°C. Immediately prior to use, the films were allowed to come to room temperature, solubilized to 5 mM in anhydrous DMSO, sonicated in a bath sonicator (Branson) for 10 minutes, diluted to 100 μM in phenol-red free Hams F12, and stored at 4°C for 24 hours. Oligomeric Aβ42 morphology was routinely confirmed by atomic force microscope [19].

### Cell culture and cell viability assay

Mouse neuroblastoma, N2A cells (ATCC) were maintained in MEM (ATCC) supplemented with 10% FBS, 2 mM L-glutamine, 100 U/ml of penicillin, 100 ug/ml of streptomycin, as previously described [18,69]. 5000 cells per well were plated on to 96-well plates 24 hours prior to treatment to allow attachment. Cells were then treated 10 mM oAβ42 in DMEM medium without phenol red and with 1% N2 supplement (Invitrogen). At the end of the experiment (24 hours post-treatment), cell viability was assessed by relative cellular ATP levels using CellTiter-Glo assay kit (Promega) according to the manufacture's instruction. Statistical significance was established at p < 0.01 by One-way ANOVA with Tukey test for comparison in different groups.

### Pharmacological inhibition of endocytosis

N2A cells were treated with pharmacological inhibitors that block specific steps during endocytosis. Pilot experiments were performed to find inhibitor concentrations that did not significantly compromise cell viability as inhibitors for cellular endocytosis could adversely affect cell viability. For example, as has been reported, chlorpromazine at higher concentration (10^-4^-10^-3 ^M) killed cells, while at lower concentration (10^-6^-10^-5 ^M) inhibited Ca^++^-mediated toxicity in a neuroblastoma cell line [93]. Genistein substantially inhibited the growth of N2A cells in a dose-dependent manner with an IC50 value of 18 mM, and PP2 at 3 mM was lethal to the N2A cells [94]. Chlorpromazine (2 mM), genistein (10 mM), PP2 (1 mM) were added to cell cultures at indicated concentrations 1 hr before oAβ42 treatment in DMEM with 1% N2 supplement. The final concentration of vehicle (DMSO) was 0.05% in all cultures.

### Genetic manipulation of selected endocytic pathway proteins

To block specific routes in the endocytic pathways, we blocked the function of key proteins in the endocytic pathway by either expressing dominant-negative proteins, or knock-down of endogenous proteins. N2A cells were transiently transfected with expression plasmids or siRNA using LipofeactAmine 2000 (Invitrogen). The following endocytic proteins were transfected for expression: Rat wild type (WT) Dynamin and the dominant-negative dynamin mutant K44A (myc-tag), the dominant-negative AP180 mutant AP180-CT (Flag-tag), and RhoA WT and dominant-negative mutant T19N (HA-tag). These plasmids were kindly provided by Dr. R. Minshall (UIC, dynamin) and Dr. L. Greene (NIH, AP180), or purchased from Missouri S&T (RhoA). To achieve highest possible transfection efficiency, we tested several transfection reagents (such as LipofectAmine and PLUS reagent, GenJet, and LipofectAmine 2000) and transfection conditions (cell density, pH of the medium and transfection incubation duration) with EGFP expression plasmid. We obtained the highest transfection efficiency with LipofectAmine 2000 at cell density of 90-100% confluence.

Small interfering RNA (siRNA) for the clathrin heavy chain (CLTC, SMARTpool L-004001-00-0005) and control Non-Targeting siRNA were purchased from Dharmacon. Cells were transfected at 20 pmol siRNA in 24-well culture plates using LipofectAmine 2000 according to vendor's recommended transfection protocol. A second transfection was done the next day. These transfected cells were then split and seeded on to 96-well plate in MEM with 10% FBS. 48 hours post-transfection, these cells were treated with oAβ42 for 24 hours.

### SDS-PAGE/Western blot characterization of targeted endocytic proteins

Transfected cells treated in parallel to those used for cell viability assays were lysed by 15-minute incubation in RIPA buffer (50 mM Tris-HCl, pH 8.0, with 150 mM sodium chloride, 1.0% Igepal CA-630 (NP-40), 0.5% sodium deoxycholate, and 0.1% SDS, Sigma-Aldrich) containing protease inhibitors (Protease Inhibitor Cocktail Set I, Calbiochem), followed by centrifugation. Equal amounts of total protein were analyzed for levels of indicated proteins by Western blot analysis following SDS-PAGE using 4-12% Bis-Tris 1.5 mm NuPAGE precast gels (Invitrogen). Supernatants were mixed with LDS sample buffer (Invitrogen) and electrophoresed at 90-100 V for 80-90 minutes. Proteins were transferred to 0.2 μm polyvinylidene difluoride membranes. Membranes were blocked for 1 hour in a solution of 5% nonfat dry milk in Tris-buffered saline containing 0.0625% Tween-20 prior to incubation with primary antibody solutions. Molecular mass was estimated using pre-stained molecular weight markers (Invitrogen). CLTC was detected using a mouse anti-CLTC monoclonal antibody (C1860, Sigma; 1:1,000), myc tagged dynamin with anti-myc antibody 9E10 (Sigma; 1:5000), Flag-tagged AP180-CT with Flag antibody M2 (Sigma, 1:5000), HA tagged RhoA with rat HA antibody 3F10 (Roche Applied Science; 1:2500), and appropriate horse radish peroxidase conjugated secondary antibody. Actin, as detected with rabbit anti-actin antibody (Sigma; 1:5000), was used as total lysate loading control. Proteins were visualized with enhanced chemiluminescence Western blotting substrate (Pierce) on the Kodak 4000R imaging system.

### Cellular uptake of oligomeric Aβ42

Intracellular Aβ was detected by immunofluorescence analysis using a rabbit polyclonal anti-Aβ42 specific antibody (Invitrogen). N2A cells or transiently transfected N2A cells (24 hours post transfection) were seeded at 20,000 cells/well on poly-D-lysine glass coverslips in phenol-red free DMEM + 10% FBS overnight. Recombinant oAβ42 was added to cells in DMEM medium and incubated for 30 min at 37°C. At the end of the treatment, cells were washed with PBS. Cell surface bound oAβ was striped off in a solution of 0.2 M acetic acid and 0.5 M NaCl. Cells were fixed in 4% paraformaldehyde for 20 minutes at room temperature. Cells were permeablized with 0.3% Triton X-100 in 1×PBS for 5 minutes, and blocked for 15 minutes with 3% BSA, incubated overnight with rabbit anti-Aβ42 (1:100) at 4°C, followed by 1 hour incubation at room temperature with Alexa488-labeled donkey-anti-rabbit IgG (1:500, Invitrogen). Transfected cells were identified by co-staining with anti-Flag antibody (M2, 1:250, Sigma) for AP180, rat anti-HA (3F10, Roche) for RhoA, or mouse anti-myc antibody 9E10 (1:200, Abcam) for dynamin, and appropriate 2^nd ^antibody conjugated with Alexa594 (all from Invitrogen). Coverslips were mounted with Prolong Gold antifade reagent with DAPI (Invitrogen) fluorescence mounting medium on glass slides. Confocal laser scanning microscopy images were acquired on a Zeiss LSM 510 META, Axiovert 200 M laser scanning confocal microscope using a Plan-Apochromate Zeiss 40×/1.3 oil immersion objective. Mean brightness of Aβ signals were quantified with NIH image software.

## Competing interests

The authors declare that they have no competing interests.

## Authors' contributions

CY designed and performed the experiments, collected and analyzed the data, and wrote the manuscript. EN and KL collected and analyzed the data. MJL contributed to experimental design and data analyses, and preparation of the manuscript. All authors read and approved the final manuscript.
